# Higher temperatures distinctly affect the brain mitochondrial respiration of two Amazonian fishes, a predator and a prey

**DOI:** 10.1007/s10695-026-01691-3

**Published:** 2026-05-07

**Authors:** Waldir Heinrichs-Caldas, Jhonatan Mota da Silva, Jefferson de Almeida Silva, Jaqueline Custódio da Costa, Maria de Nazaré Paula Silva, Adalberto Luis Val

**Affiliations:** Laboratório de Ecofisiologia E Evolução Molecular (LEEM), Instituto Nacional de Pesquisas da Amazônia, Campus I, Manaus, Amazonas Brazil

**Keywords:** Amazon basin, Piranha, Aracu, Mitochondria, Temperature

## Abstract

The aquatic biota of the Amazon is subject to considerable daily and seasonal fluctuations in its environment. The ability to adapt to changes in the environment, such as increased water temperatures, is essential for the survival and physiological performance of these organisms. The anticipated rise in temperature and CO_2_ levels, projected to reach 6 °C and 50% above current levels by the end of the century, respectively, represents a significant and imminent threat to aquatic biodiversity. The objective of this study was to assess the effects of acute short-term exposure to elevated temperature on the brain mitochondrial respiration of two Amazonian fish species with contrasting trophic roles: *Schizodon fasciatus*, which represents a prey species at a lower trophic level, and *Pygocentrus nattereri*, a predatory species. Specimens of *Schizodon fasciatus* and *Pygocentrus nattereri* were collected and subjected to temperatures of 28 °C and 37 °C for a period of four hours. Brain mitochondrial respiration was quantified to analyze complexes I, I + II, III, IV, proton leak (H^+^Leak), RCR, and ETS. Additionally, the production of reactive oxygen species (ROS) was evaluated. The results revealed that exposure to higher temperatures caused elevated complex I activity in both species, with a more pronounced increase observed in *S. fasciatus*. Furthermore, significant increases in complex I + II and complex III were observed in *S. fasciatus*, while complex IV showed a decrease in both species. H^+^Leak exhibited a decrease in *P. nattereri*, whereas RCR demonstrated an increase in both species. ROS production displayed a decrease in *P. nattereri* but remained stable in *S. fasciatus*. The findings suggest that both fish species possess the capacity to adapt their brain mitochondrial function to higher temperatures, albeit with species-specific responses. These adaptations assist in maintaining brain energy production and minimizing oxidative stress, and emphasize potential imbalances in predator–prey relationships under changing environmental conditions.

## Introduction

The aquatic biota of the Amazon is subject to a number of environmental variations on a daily and seasonal basis. The ability of aquatic organisms to adapt to environmental fluctuations, such as alterations in water temperature, is vital for their survival and proper physiological functioning. As projected by the Intergovernmental Panel on Climate Change (IPCC), by the end of the century, in an extreme scenario, the temperature may increase by 6 °C, along with an increase in 850 ppm of CO_2_, which may result in new threats to aquatic diversity (IPCC 2021). The Amazon basin is already exhibiting some effects, including an increase in water temperature. In 2023, a drought of unprecedented severity led to water temperatures reaching 40 °C, resulting in mass mortality of aquatic biota and significant disruption to the ecosystem’s trophic chain (Wilen and Wilen 2012; Espinoza et al. 2024).

Temperature, dissolved oxygen, nutrients, and light, among other abiotic factors, frequently serve as mediators of trophic interactions (Guerrero‐Ramírez and Eisenhauer 2017). Because temperature can alter trophic interactions and organismal performance, species occupying different trophic roles may not respond similarly to warming (Gibert 2019). The consumer-resource dynamic (Amarasekare 2015), herbivorous-plant interaction (O'Connor 2009), and overall trophic dynamics can be negatively impacted by higher temperatures (Gilbert et al. 2014). Understanding whether predator and prey fishes differ in their physiological responses to acute thermal stress may therefore help clarify how climate extremes could affect ecological interactions.

Fish rely on external heat sources to regulate their body temperature, which is contingent upon the temperature of their surrounding environment. It is possible that alterations to thermal habitats may affect not only species that are sensitive to temperature changes, but also those that are more resistant, at levels ranging from the molecular to the ecological level (Payne et al. 2021). This could potentially result in an imbalance within food webs. For example, alterations in temperature that result in the absence of a top predator may lead to changes in the abundance of lower trophic levels (Kishi et al. 2005). This is because the abundance and type of predators regulate trophic linkages. In other words, when one component of the food web is threatened, the integrity of the entire system is compromised.

Campos et al. (2021) demonstrated that fish species that are native to South America already inhabit environments with temperatures that approach their upper thermal limits. To withstand elevated temperatures, Amazonian fish undergo a range of adaptations, from genetic to metabolic and respiratory adaptations (Heinrichs-Caldas et al. 2019; Val and Almeida-Val 1995). These changes in ATP demand and utilization are linked to mitochondrial respiratory control (Bertram et al. 2006). Consequently, when temperature exposure is a limiting factor, cell homeostasis is disrupted, mitochondrial metabolism is affected, and cellular bioenergetics and redox balance are altered in different tissues, such as the brain (Sokolova 2023).

The physiology and functioning of the brain are significantly affected by elevated temperatures (Pörtner 2012; Pörtner et al. 2017). Changes in brain physiology impact the cognitive abilities of fish (Toni et al. 2019), their reproductive capacity (Servili et al. 2020), their developmental processes (Beltrán et al. 2021), and their behavioral patterns (Nonnis et al. 2021). These changes can potentially disrupt the equilibrium of natural trophic webs. The brain’s production of energy necessitates a substantial quantity of oxygen to sustain its metabolic activities, with mitochondrial respiration serving a pivotal role in regulating this process (Picard and McEwen 2018). Mitochondrial respiration is fundamental for the production of cellular energy and is crucial in the response of species to thermal changes (Johnston et al. 1994). However, comparative information remains limited for Amazonian freshwater fishes, particularly regarding how species occupying contrasting trophic roles adjust brain mitochondrial function under acute thermal stress.

In the present study, we used two Amazonian freshwater fishes with contrasting trophic roles: *Schizodon fasciatus* (Spix and Agassiz, 1829), considered here as the prey species, and *Pygocentrus nattereri* (Kner, 1858), the predator species. *Schizodon fasciatus* occupies a lower trophic position and is frequently exposed to intense environmental variation in floodplain systems, whereas *P. nattereri* is a predatory characiform whose ecological performance depends on the maintenance of sensory and locomotor capacities associated with prey detection and capture. Because predator and prey species may differ in energetic demand, mitochondrial plasticity, and vulnerability to thermal stress, comparing these two fishes provides a relevant framework for testing whether acute warming elicits distinct brain mitochondrial responses in species occupying contrasting ecological roles.

Therefore, in this study, we investigated the effects of acute exposure to elevated temperature on brain mitochondrial respiration (complexes I, I + II, III, IV, proton leak, RCR and ETS) and total reactive oxygen species (ROS) in the brain of *Schizodon fasciatus*, a prey species, and *Pygocentrus nattereri*, a predator species, in order to assess whether species occupying contrasting trophic roles show distinct physiological responses to thermal stress. We hypothesized that the predator *Pygocentrus nattereri* and the prey *Schizodon fasciatus* would differ in their brain mitochondrial responses to acute thermal stress, reflecting species-specific bioenergetic adjustments associated with their contrasting trophic roles and ecological demands. These species were selected because they represent contrasting trophic roles in Amazonian freshwater ecosystems and provide a biologically relevant comparison to test whether acute warming elicits species-specific mitochondrial adjustments in the brain.

## Materials and methods

### Experimental fish

Animals were obtained using monofilament gillnets of standardized dimensions in lakes near the Solimões River (S 03°20′57.3″ W 060°11′47.2″, S 03°22′58.5″ W 060°13′43.5″, S 03°23′04.6″ W 060°13′56.5″, S 03°24′29.4″ W 060°15′18.4″) and brought to a floating house, near the boat Ana Clara, for a four hour recovery and stabilization period after capture and handling, prior to the experimental exposure. Fish were kept in 500 L tanks with circulating water, directly from the river, until the beginning of the experiment. During the capture of the fish, the water temperature and oxygen concentration were 29.3 °C ± 0.8 and 5.33 mgO_2_ L^−1^ ± 0.3, respectively. The capture of the animals, their transport, sampling, care, and the experiment itself were performed after permission was obtained from SISBio (#29,837—24), CEUA/INPA: 004/2018 INCT ADAPTA II.

### Acute *in vivo* exposure

For the experiment, *S. fasciatus* (45.8 g ± 3.9) and *P. nattereri* (75 g ± 6.2) were placed in individual 5-L aquariums and each species was subjected to temperatures of 28 °C (*n* = 6) and 37 °C ± 0.02 (*n* = 6) for four hours. Prior to the exposition, the animals were kept in the individual aquariums for 2 h to recovery from handling. The higher temperature was selected to correspond to the highest temperature recorded in the Solimões River during the expedition. For the 37 °C treatment, water temperature was gradually increased at a rate of 0.2 °C min^−1^ using a Roxin Q3 thermostat heater (50 W, 110 V) until the target temperature was reached. Once established, the temperature was maintained stable by the thermostat during the entire 4—h exposure. Water temperature and dissolved oxygen were continuously monitored with an Oximeter 5512-FT (YSI, Yellow Springs, OH, USA). Following the conclusion of the exposure period, the animals were euthanized via a combination of head concussion and a cut to the spinal cord. Subsequently, the brain was excised and stored in BIOPS buffer (2.77 CaK_2_EGTA, 7.23 K_2_EGTA, 5.77 Na_2_ATP, 6.56 MgCl_2_.6H_2_O, 20 taurine, 20 imidazole, 0.5 dithiothreitol, 50 K-MES, 15 sodium phosphocreatine, all measured in mM) for immediate analysis of mitochondrial respiration.

### Mitochondrial respiration

All the samples were analyzed on the same day that they were collected and all mitochondrial respiration measurements were performed at 28 °C in the Oroboros O_2_K. Brains were weighted (20.08 mg ± 0.03) and cut into pieces of approximately 2 mm^3^ using small sterilized scissors and sharp forceps, and immersed in ice cold BIOPS until the analyses. After this, the brains were dried and immersed in 0.5 mL of ice cold MiR05 buffer (pH 7.24): 0.5 EGTA, 3.0 MgCl_2_·6H_2_O, 60 potassium lactobionate, 20 taurine, 10 KH_2_PO_4_, 20 Hepes, 160 sucrose (all in mM), and BSA 1.0 g/L, essentially fatty acid free, and homogenized by hand. This homogenate was then diluted in 2.0 mL MiR05 in order to measure the oxygen consumption in an oxygraph (O_2_K Oroboros, Innsbruck, Austria) (Gnaiger et al. 2000). During the whole process, the samples and solutions were kept in the ice. Respiratory flux through complexes I, II, III, and IV of the electron transport chain was measured with the software OROBOROS DatLab 6.

For the mitochondrial respiration, we used the SUIT protocol, with total volume of 2.0 mL per chamber. The oxygen was calibrated prior the assays, In order to measure complex I (CI) state II respiration, 2 mM malate and 10 mM pyruvate were added to the chambers to measure CI without ADP. Later, 2.5 mM ADP were added to stimulate oxidative phosphorylation (OXP-I), and 10 mM glutamate were added to saturate CI. Cytochrome c (10 μM) was added in order to assess mitochondrial membrane integrity. Phosphorylating respiration with CI and complex II (CII) substrates (CI + II) was measured after the addition of 10 mM succinate. The ETS, respiratory electron transfer-pathway capacity, was measured by the addition of carbonyl cyanide p-(trifluoromethoxy) phenyl-hydrazone (0.5 mM FCCP). The activity of complexes CI, II, and III was then inhibited by the addition of rotenone (0.5 μM), malonate (15 mM), and antimycin (1 μM), respectively. RCR was calculated as ETS/leak state ratio. In order to activate the complex IV (CIV) respiration, the terminal oxidase of the mitochondrial ET-pathway, 2 mM ascorbate and 0.5 mM TMPD (N,N,N′,N′-tetramethyl-p-phenylenediamine dihydrochloride) were added to the mitochondria medium. Respiration rates are expressed as pmol O₂ s⁻^1^ mg⁻^1^ fresh tissue.

Total ROS production was measured alongside the mitochondrial respiration. SOD of 22.5 U.mL^−1^ was added to catalyze the reaction of the superoxide produced by the mitochondria and 3 U mL^−1^ horseradish peroxidase was added to catalyze the reaction of hydrogen peroxide with 15 μM Amplex UltraRed, which results in the release of resorufin, a fluorescent substance. The equipment detects the resorufin using a wavelength of 525 nm and an ampliometric filter set (AmR, Oroboros Instruments). The resorufin signal was calibrated with additions of exogenous hydrogen peroxide.

### Statistics

Data are presented as mean ± SEM. For each response variable, we used a two-way ANOVA with species (*Schizodon fasciatus* and *Pygocentrus nattereri*) and exposure temperature (28 °C and 37 °C) as fixed factors, followed by Tukey’s post hoc test when appropriate. The interaction between species and temperature was also evaluated. Assumptions of normality and homogeneity of variances were checked prior to analysis. The statistical analyses were performed in SigmaStat (v. 3.5) and the graphs were created using SigmaPlot software (v. 11.0).

## Results

**Fig. 1:**
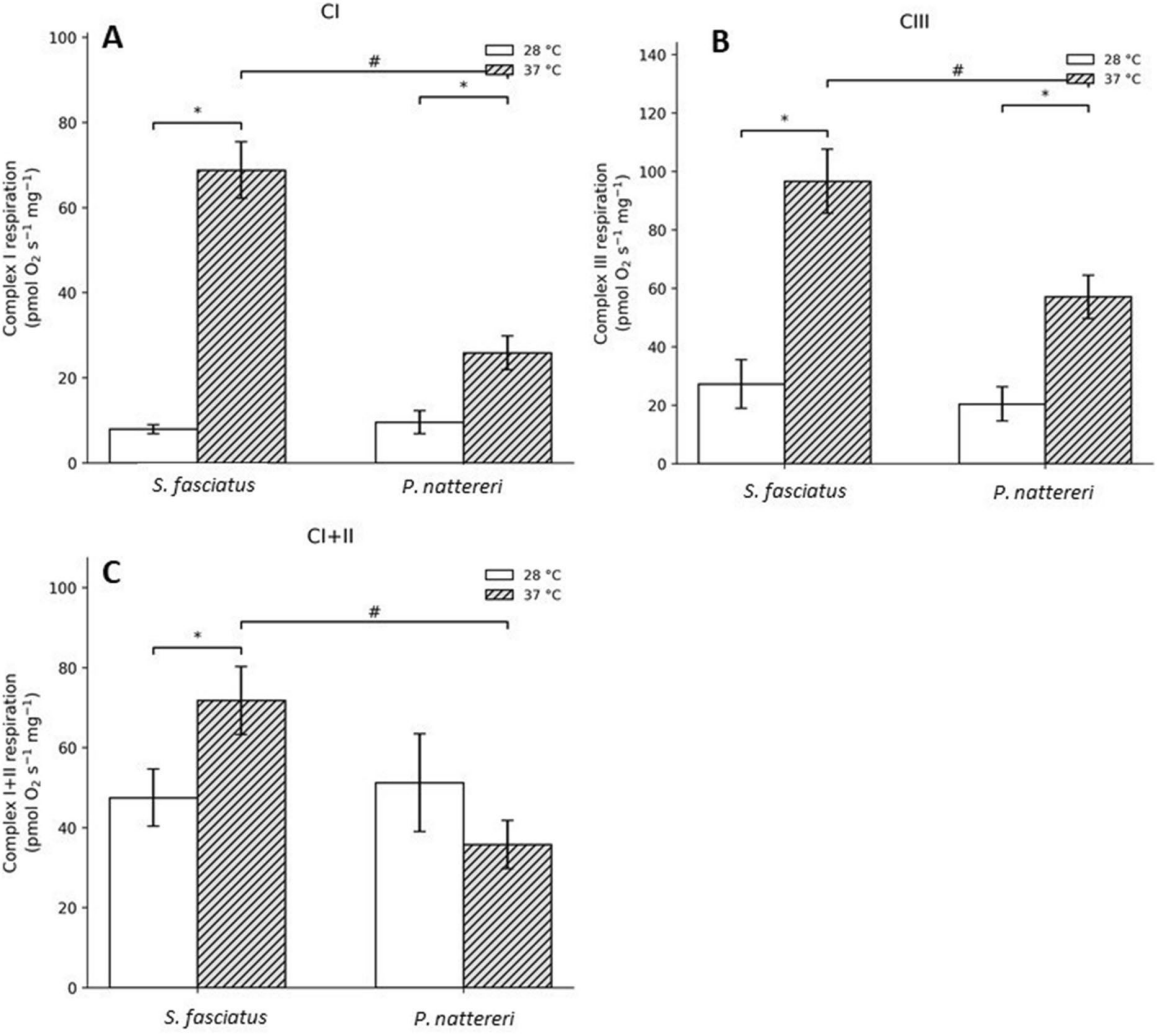
Brain mitochondrial respiration in *Schizodon fasciatus* and *Pygocentrus nattereri* after acute exposure to 28 °C or 37 °C for 4 h. Panels show complex I (CI) (**A**), complex I + II (CI + II) (**B**), and complex III (CIII) (**C**). Bars represent mean ± SEM. White bars indicate 28 °C and gray hatched bars indicate 37 °C. Asterisks (*) indicate significant differences between temperatures within the same species, whereas number signs (#) indicate significant differences between species at 37 °C (two-way ANOVA followed by Tukey’s post hoc test, *p* < 0.05)

**Fig. 2:**
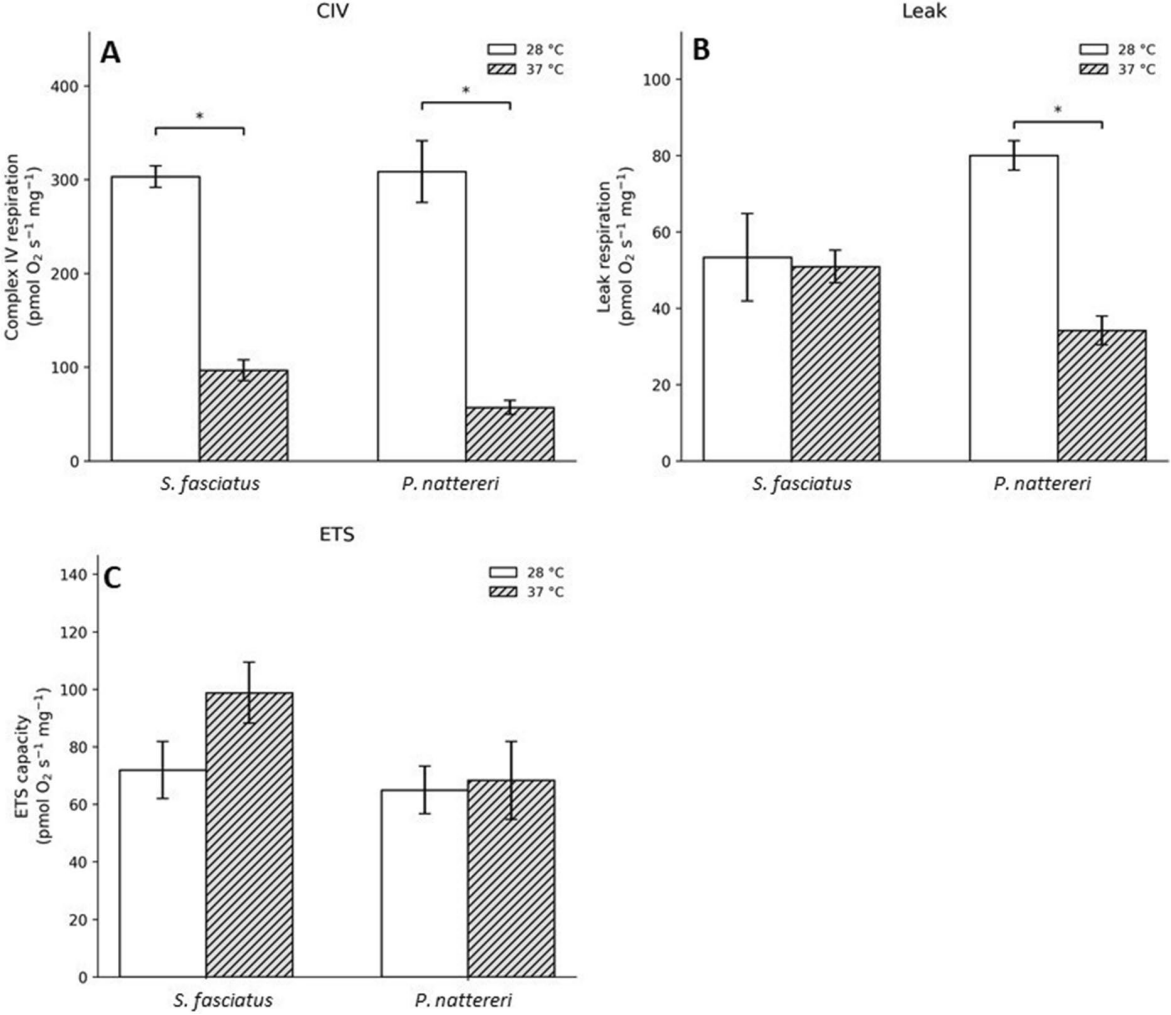
Brain mitochondrial respiration in *Schizodon fasciatus* and *Pygocentrus nattereri* after acute exposure to 28 °C or 37 °C for 4 h. Panels show Complex IV (CIV) (**A**), proton leak (Leak) (**B**), and electron transport system capacity (ETS) (**C**). Bars represent mean ± SEM. White bars indicate 28 °C and gray hatched bars indicate 37 °C. Asterisks (*) indicate significant differences between temperatures within the same species (two-way ANOVA followed by Tukey’s post hoc test, *p* < 0.05). No significant differences were detected for ETS

**Fig. 3:**
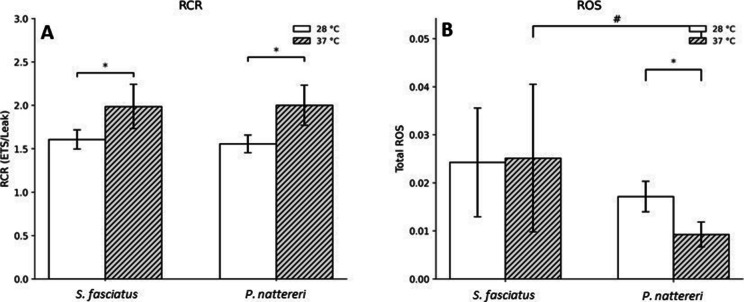
Coupling efficiency and oxidative status in brain mitochondria of *Schizodon fasciatus* and *Pygocentrus nattereri* after acute exposure to 28 °C or 37 °C for 4 h. Panels show respiratory control ratio (RCR) (**A**) and total reactive oxygen species production (total ROS) (**B**). Bars represent mean ± SEM. White bars indicate 28 °C and gray hatched bars indicate 37 °C. Asterisks (*) indicate significant differences between temperatures within the same species, whereas number signs (#) indicate significant differences between species at 37 °C (two-way ANOVA followed by Tukey’s post hoc test, *p* < 0.05)

All analyses were performed aboard the Ana Clara during the expedition, demonstrating the feasibility of obtaining high-resolution mitochondrial respiration measurements under field conditions in the Amazon. Acute exposure to 37 °C altered brain mitochondrial respiration in both species, although the magnitude and direction of the responses differed between *S. fasciatus* and *P. nattereri*. Complex I respiration was significantly affected by species and temperature (two-way ANOVA, *p* < 0.001, *F* = 29.113; Fig. 1A). Both species showed higher complex I respiration after exposure to 37 °C compared with 28 °C, with a more pronounced increase in *S. fasciatus* than in *P. nattereri*. Complex I + II respiration also differed between species and temperatures (Fig. 1B). In *S. fasciatus*, exposure to 37 °C increased complex I + II respiration relative to 28 °C (*p* = 0.038, *F* = 5.123), whereas values at 37 °C were higher in *S. fasciatus* than in *P. nattereri* (*p* = 0.011). Complex III respiration was significantly affected by both temperature and species (two-way ANOVA, *p* < 0.001, *F* = 40.378; Fig. 1C). Both species showed higher complex III respiration at 37 °C, but the increase was greater in *S. fasciatus* than in *P. nattereri* (*p* = 0.014). In contrast, complex IV respiration decreased markedly in both species after exposure to 37 °C (two-way ANOVA, *p* < 0.001, *F* = 151.921; Fig. 2A). This reduction was observed in both *S. fasciatus* and *P. nattereri*. Proton leak was also affected by species and temperature (two-way ANOVA, *p* = 0.005, *F* = 10.505; Fig. 2B), with a significant decrease observed in *P. nattereri* at 37 °C (*p* < 0.001), whereas no significant change was detected in *S. fasciatus*. In contrast, ETS capacity did not differ significantly between species or temperatures (two-way ANOVA, *p* = 0.290, *F* = 1.196; Fig. 2C). Respiratory control ratio (RCR) increased in both species after exposure to 37 °C (two-way ANOVA, *p* = 0.043, *F* = 4.850; Fig. 3A), indicating increased mitochondrial coupling efficiency under acute warming. Total ROS production differed between species and temperatures (two-way ANOVA, *p* = 0.009, *F* = 122.90; Fig. 3B). *Pygocentrus nattereri* showed lower ROS production overall than *S. fasciatus* (*p* = 0.001), and ROS levels further decreased in *P. nattereri* after exposure to 37 °C (*p* = 0.039). In contrast, ROS production in *S. fasciatus* remained unchanged between temperatures.

## Discussion

### Acute warming and brain mitochondrial adjustments

The objective of this study was to compare the effects of acute thermal exposure on brain mitochondrial function in two species that are related through a trophic web: *P. nattereri*, a predator, and *S. fasciatus*, the prey. The findings of this study indicate that both species can alter mitochondrial brain respiration at temperatures approaching their limits, with modifications that are specific to each species (Table 1). These modifications facilitate the maintenance and protection of brain energy production. Although mitochondrial respiration is closely associated with an animal’s thermal tolerance, mitochondria often exhibit thermal limits that exceed those of the entire organism. This suggests that heat-induced damage to mitochondria is unlikely to be the primary cause of death in these animals, although it may disrupt functions including those of the nervous system (Martinez et al. 2016; Chung and Schulte 2020).

**Table 1:** Summary of the main brain mitochondrial responses of *Schizodon fasciatus* and *Pygocentrus nattereri* after acute exposure to 37 °C for 4 h, relative to their respective controls at 28 °C. Arrows indicate the direction of change under warming, and the final column summarizes the main comparative pattern observed between species

Variable	*S. fasciatus*	*P. nattereri*	Comparative pattern
CI	↑	↑	Greater increase in *S. fasciatus*
CI + II	↑	No change/lower	Higher at 37 °C in *S. fasciatus*
CIII	↑	↑	Greater increase in *S. fasciatus*
CIV	↓	↓	Similar directional decrease
Leak	No change	↓	Lower leak in *P. nattereri* at 37 °C
ETS	No change	no change	Stable in both species
RCR	↑	↑	Increased coupling in both
ROS	No change	↓	Lower ROS in *P. nattereri*

### Differences between species in mitochondrial responses

Mitochondrial oxidative phosphorylation (OXPHOS) is responsible for regulating cellular energy production. It has been demonstrated that elevated temperatures can have a detrimental effect on this process (Sokolova 2018; Gerber et al. 2020). In the process of oxidative phosphorylation, the function of complex I is of particular importance due to it generating an electrochemical proton gradient across the inner mitochondrial membrane (Sharma et al. 2009). While complex II plays an essential role in electron transfer and the production of FADH_2_ from succinate (Bezawork-Geleta et al. 2017), both complexes, along with complex III, which transfers electrons from ubiquinone to cytochrome c while pumping protons, contribute to ATP production (Chandel 2010). Complex IV is responsible for reducing molecular oxygen to form water, releasing protons in the mitochondrial matrix and contributing to the generation of the electrochemical gradient necessary for ATP synthesis (Kadenbach 2021).

The findings reveal that both fish species exhibited an elevation in mitochondrial complex I activity in response to elevated temperatures. However, *S. fasciatus* exhibited a more pronounced increase in complex I activity compared to the *P. nattereri*. Additionally, *S. fasciatus* demonstrated an increase in mitochondrial complex I + II, whereas *P. nattereri* exhibited a decrease in this complex. Both species demonstrated an increase in mitochondrial complex III, with *S. fasciatus* exhibiting a more pronounced increase. The results demonstrate that, despite the elevated temperature, there is no collapse in aerobic metabolism in the brains of either species. It is proposed that this increase in mitochondrial respiration is associated with the level of activity exhibited by these species. *Schizodon fasciatus* and *P. nattereri* are noted for their high level of activity, including migration, and are constantly engaged in foraging, hunting and other activities that require a substantial consumption and delivery of oxygen and ATP demand (Mérona and Rankin-de-Mérona 2004; Ferreira et al. 2014). Consequently, these species rely on aerobic metabolism to meet their energy needs.

Our findings align with those of previous studies on *Pterygoplichthys pardalis* (Lima et al. 2025) and *Salmo salar* (Gerber et al. 2020), and indicate that an acute elevation in temperature enhances mitochondrial energy production in the brains of our two species. Additionally, our findings show that, in the brains of *S. fasciatus*, the activities of complexes I, II, and III appear to be complementary, as evidenced by an increase in the activity of one complex relative to the others, which differs from our observations of what occurred in *P. nattereri*. No decrease in OXPHOS complexes was observed in either of the species, indicating that the animals’ mitochondria were not approaching their thermal limits. The observed increases in complex I and, in *S. fasciatus*, Complex I + II indicate that acute exposure to 37 °C did not cause an immediate collapse of brain mitochondrial function under the conditions tested. Therefore, the temperature used in this study and the duration of the exposure were insufficient to disrupt the mitochondrial Arrenius plots (Sokolova 2023), as evidenced by the observed increase in the OXPHOS complexes for both species, which were not manifested in the H^+^Leak, thus indicating the absence of mitochondrial respiration collapse.

In contrast to the other complexes, both species demonstrated a reduction in mitochondrial complex IV activity in response to their exposure to higher temperature. Complex IV is fundamental for cellular energy production during aerobic respiration (Kadenbach 2021). The decrease in complex IV observed in both species indicates that the electron flux may not be allocated to ATP synthesis, which is consistent with the results observed with ETS. According to the method applied here to calculate ETS (SUIT-protocol), there is maintenance of energy production, yet both species show no increase.

The mitochondrial ETS is responsible for the transfer of electrons from the donor to the final electron acceptor, while simultaneously pumping protons to create an electrochemical gradient that is utilized for ATP synthesis. As a result, the ETS plays a central role in cellular energy production. The literature indicates that ETS responses to temperature are species and tissue specific (Iftikar et al. 2015; Du et al. 2016; Heinrichs-Caldas and Almeida-Val 2021). Despite the expectation that an increase in ETS would occur in response to warming, neither species demonstrated differences in brain mitochondrial ETS at the two temperatures. This finding is consistent with the direct relationship observed between ETS and total ROS, a topic that will be discussed in more detail.

### ROS, leak, and coupling efficiency under thermal stress

Nevertheless, it is anticipated that elevated temperatures enhance the overall mitochondrial electron flux rate in the LEAK state, consequently reducing the RCR (Yan and Xie 2015). Our findings indicate that, in the case of *P. nattereri*, the decline in H^+^Leak is associated with an uptake in RCR, which then gives way to a decline in total ROS production. The state of mitochondrial leakage may have significant implications for cellular function and energy metabolism (Jastroch et al. 2010). In certain circumstances, a certain degree of mitochondrial leakage may be adaptive, facilitating the dissipation of excess energy or the reduction of oxidative stress (Munro and Treberg 2017). However, when present in excess, it can contribute to decreased energy efficiency and oxidative stress, which is associated with several pathological conditions, including metabolic, neurodegenerative and cardiovascular diseases (Salim 2017; Menon et al. 2023).

The results for *S. fasciatus* showed no difference in H^+^Leak, and *P. nattereri* demonstrated a decrease. Despite this, both fish species exhibited an increase in the RCR, a measure of mitochondrial coupling efficiency, in response to the higher temperatures. An elevated RCR is indicative of optimal mitochondrial function, whereas a reduced RCR suggests mitochondrial dysfunction (Rolfe, and Brown 1997; Brand and Nicholls 2011). Any alterations in oxidative phosphorylation will consequently result in changes to the RCR. Notably, *P. nattereri* exhibited a decrease in mitochondrial H^+^Leak, which signifies a decline in mitochondrial efficiency, as evidenced by the reduction in mitochondrial ROS production observed in this species. The increase in RCR for both species also indicates that the tissue did not reach its threshold when exposed to 37 °C.

Modifications in the overall ROS level give rise to imbalances in ROS generation and consumption, which in turn give rise to imbalances in the mitochondrial energy economy and activity (Van der Oost et al. 2003). The reduced ROS levels observed in *P. nattereri* may be associated with mitochondrial uncoupling in this species, as well as a decline in H^+^Leak, suggesting that ROS production may be more active during H^+^Leak in this species (Munro and Treberg 2017). The reduction in total ROS observed in *P. nattereri* may also be attributed to the decline in complex IV, which diminishes the proton motive force available for ATP production (McDonald et al. 2009).The mitochondrial activity of the entire brain in *P. nattereri* indicates that energy is allocated to protect the cell against oxidative stress in response to warming. In contrast, the brain mitochondria of *S. fasciatus* may follow different pathways that permit the maintenance of ROS levels, including antioxidant activity. Overall, the maintenance of ETS is a common feature of both species.

### Potential ecological implications for predator–prey interactions

The present results indicate that both species were able to maintain brain mitochondrial function during acute warming, but through distinct physiological adjustments. In *S. fasciatus*, the marked increase in complex I, complex I + II, and complex III, together with stable ROS levels, suggests a stronger upregulation of mitochondrial electron flow in response to thermal challenge. In contrast, *P. nattereri* showed a more moderate increase in respiratory complexes, accompanied by reduced proton leak and lower ROS production, which may indicate a more conservative mitochondrial adjustment under acute heat stress. Species-specific mitochondrial responses to warming have been described in fishes and other ectotherms, reinforcing the idea that thermal stress does not affect energy metabolism uniformly across taxa or tissues (Iftikar et al. 2015; Gerber et al. 2020; Lau et al. 2020; Sokolova 2023). Such differences may reflect variation in metabolic demand, mitochondrial plasticity, and the capacity to balance ATP production with oxidative protection (Sokolova 2018; Chung and Schulte 2020).

These interspecific differences may also have ecological relevance. Temperature and CO_2_ are known to affect fish behavior, sensory performance, foraging, and predator–prey interactions, potentially disrupting ecological relationships under climate change (Munday et al. 2009; Nowicki et al. 2012; Pörtner et al. 2017). In this context, the distinct mitochondrial responses observed here in the brains of *S. fasciatus* and *P. nattereri* suggest that acute warming may affect the physiological basis underlying ecological performance differently in prey and predator species. Although our study did not directly measure behavior or predation success, the differential mitochondrial adjustments observed between species support the hypothesis that thermal extremes may contribute to imbalances in trophic interactions in Amazonian aquatic systems.

In *S. fasciatus*, the increase in Complexes I, I + II, and III, together with the decrease in complex IV, suggests a strong mitochondrial response to acute warming that may help sustain ATP production in the short term but could also indicate a potential constraint on electron transport under more prolonged exposure. In contrast, *P. nattereri* showed a more moderate increase in respiratory complexes, combined with reduced H + Leak and lower ROS production, which may reflect a more conservative mitochondrial adjustment under acute thermal stress.

Ecologically, these differences may be relevant because predator and prey species do not necessarily experience the physiological consequences of warming in the same way. If these mitochondrial adjustments are linked to organismal performance, acute thermal extremes could alter the balance between prey responsiveness and predator stability. However, because our study did not directly measure behavior, escape performance, or predation success, these ecological implications should be considered as hypotheses to be tested in future studies rather than demonstrated outcomes.

## Conclusion

By using permeabilized brain tissue to access mitochondrial respiration, the results observed in this work provide clearer answers on how the organism as a whole would react to warmer temperatures. Our findings contribute to the existing knowledge regarding the function of brain mitochondria and the thermal tolerance of the animals. They highlight the importance of delivering oxygen and, consequently, maintaining energy production in the animal brain while protecting it from oxidative damage. Our results demonstrate the crucial role of this tissue in maintaining ecological functions in fish. The differential responses observed between *S. fasciatus* and *P. nattereri* underscore the necessity of considering the distinctive physiological adaptations of diverse organisms when investigating the impacts of environmental stressors, such as temperature, on their energy metabolism and oxidative stress mechanism.

Furthermore, this study underscores the necessity of elucidating how the interrelationship between prey and predator may evolve in response to the projected rise in temperature. Although our results demonstrate the capacity of both species to respond to high temperatures for four hours, the differentiated mitochondrial respiration responses under higher temperatures suggest that *P. nattereri* may be better adapted to cope with warmer conditions, potentially resulting in increased predatory pressure on *S. fasciatus*. This could lead to changes in the structure of the aquatic community, with possible consequences for the ecological dynamics and stability of Amazonian ecosystems. Furthermore, it remains unclear how the effects of climate change will affect both species and, consequently, their relationship in the long term, given the extremes currently experienced in the Amazon basin. Future studies should investigate whether these short-term mitochondrial adjustments persist under longer exposures and whether they translate into changes in behavior, performance, and predator–prey dynamics under realistic climate-change scenarios. Furthermore, this study underscores the potential impact of climate change on the aquatic biota in the Amazon and the food web; more studies are necessary, on a larger scale, to more fully comprehend this problem in the region.

## Data Availability

No datasets were generated or analysed during the current study.
